# Vaccination with cationic liposome-encapsulated CD4 and CD8 T cell neoepitopes induces superior tumor control

**DOI:** 10.1016/j.mtbio.2026.103103

**Published:** 2026-04-05

**Authors:** Felicia S. Spitzer, Jeroen Heuts, Brett J. Hos, Stefan Romeijn, Marcel G.M. Camps, Ferry Ossendorp, Koen van der Maaden

**Affiliations:** a Department of Immunology, Leiden University Medical Center, Leiden, The Netherlands; b Division of BioTherapeutics, Leiden Academic Centre for Drug Research (LACDR), Leiden University, Leiden, The Netherlands

## Abstract

Therapeutic cancer vaccines represent a promising new development in the field of cancer immunotherapy, aiming to elicit T cell responses targeting tumor antigens. Cancer vaccines consisting of multiple tumor-specific T cell peptide epitopes require optimal formulation, as peptides by themself are poorly immunogenic. In this study, we report the development of a novel multi-epitope cationic liposomal cancer vaccine utilizing real neoepitopes and evaluated the efficacy in a murine colorectal cancer model for personalized vaccination. MHC class I and class II neoepitope peptides were successfully individually encapsulated into cationic liposomes. Upon intradermal vaccination significantly higher neoepitope-specific CD8 and CD4 T cells were induced, compared to vaccination with soluble peptides or peptides mixed with empty liposomes. Moreover, this liposomal neoepitope vaccine was highly effective in preventing MC38 tumor outgrowth and cured 60% of mice bearing a lethal tumor. In conclusion, this study shows that cationic liposomal multi-epitope peptide formulations are a promising strategy for personalized cancer immunotherapy.

## Graphical abstract

**Figure:**
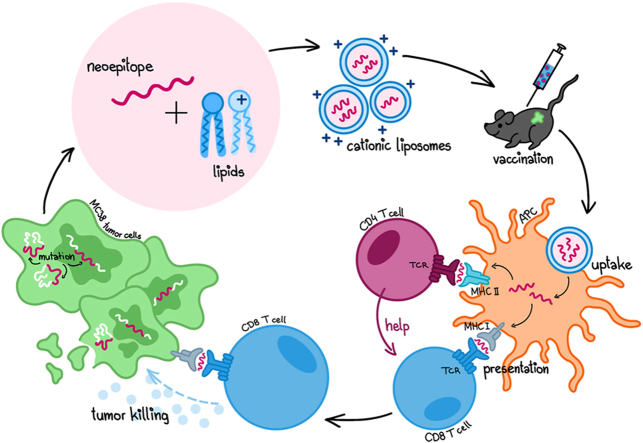

## 1 Introduction

Cancer vaccines aim to induce highly-specific anti-tumor immune responses through the activation and expansion of tumor-specific T cells, which recognize and destroy cancer cells. This T cell-mediated tumor killing relies on the recognition of tumor-specific peptide epitopes on the surface of tumor cells in the context of MHC class I and class II molecules [[1], [2], [3], [4], [5]]. Multiple clinical studies have evaluated the use of processing-dependent long synthetic peptides (SPs) containing MHC class I and class II-presented T cell epitopes as vaccines to induce polyfunctional, antigen-specific T cell responses against cancer [[6], [7], [8]]. Peptide-based vaccines facilitate highly-specific targeting of antigens, favor the formation of long-term immune memory, are cost-efficient and relatively easy to produce in GMP, are produced without the requirement of cell- and organism based systems, and are considered safe and very stable over time [9,10]. Despite the clinical potential of peptide vaccines, peptides by themselves are poorly immunogenic due to limited uptake by dendritic cells (DCs), lack of maturation signals, and consequently low antigen-specific T cell priming capacity. Therefore, peptide-based vaccines require the addition of potent adjuvants and/or formulation in delivery vehicles in order for them to induce adequate immune responses [[11], [12], [13], [14]]. Several clinical trials investigating adjuvanted peptide-based vaccines for the treatment of different cancers show promising outcomes. The most commonly used adjuvant in these vaccines is Montanide ISA-51, which improves immune responses but also comes with adverse effects, leaving room for improvement [[15], [16], [17]].

A promising alternative approach to adjuvants such as Montanide is the formulation of peptide-based vaccines in liposomes. Previously, our research group has developed a cationic liposome-based vaccination platform to efficiently induce potent antigen-specific CD8 and CD4 T cell responses [11,18]. In different murine tumor models, ovalbumin (OVA)-derived and human papillomavirus (HPV)-derived peptide epitopes encapsulated in cationic liposomes adjuvanted with TLR3 ligand Poly(I:C) were able to induce strong T cell responses that led to the clearance of fully established tumors in 75-100% of the vaccinated mice [11]. These studies also underlined the relevance of including CD4 T helper epitopes for vaccination, demonstrating that they vastly improved the efficacy of cancer vaccines compared to vaccines only comprising CD8 T cell epitopes. Further, the liposome-formulated peptide vaccine was effective even at a 65-fold lower dose compared to vaccination with unencapsulated peptide emulsified in the clinically used Montanide oil-in-water formulation, demonstrating the potency of our cationic liposomal formulations.

Currently, cancer (immuno-)therapy is shifting towards personalized treatments, for cancer vaccination this can be achieved by developing neoepitope vaccines. Neoepitopes are peptide sequences recognized my immune cells. These epitopes derive from neoantigens, which arise from random somatic DNA mutations unique to the tumor cells of a single patient [3,4]. Thereby, they are truly individual and excellent targets for personalized cancer vaccines with minimal chances of adverse effects that result from non-specific targeting [19,20]. However, the downside of this uniqueness is that each antigen has different physicochemical properties (i.e., isoelectric point and hydropathy index), which complicates the development neoepitope-based liposomal vaccine formulations. To enable production of personalized cancer vaccines encapsulating multiple, molecularly distinct peptide epitopes we have developed a liposomal formulation platform, which was evaluated for different modified ovalbumin-derived peptides [12].

This is the first study where we successfully applied our liposomal formulation platform to encapsulate multiple peptide neoepitopes into cationic liposomes and show superior tumor control. We selected two dominant tumor-derived CD8 T cell neoepitopes (*mRpl18* and *mAdpgk*) [21,22] and three recently identified CD4 T cell neoepitopes (*mDdr2, mZmiz1* and *mPcdh18*) [23] from the genetically and immunologically well-described murine colorectal cancer model MC38-L [24]. These MHC class I and class II neoepitopes were individually encapsulated utilizing our platform, which resulted in liposomal formulations with comparable physicochemical characteristics. Furthermore, prophylactic vaccination with these formulations induced functional neoepitope-specific CD8 and CD4 T cells, and protected mice against a lethal tumor challenge. Moreover, in a therapeutic setting where mice were first challenged with a lethal tumor, 60% of mice were cured upon receiving our cationic liposomal multi-neoepitope peptide vaccine.

## 2 Results

### 2.1 Characterization of cationic liposomes loaded with synthetic peptide neoepitopes

**Fig. 1:**
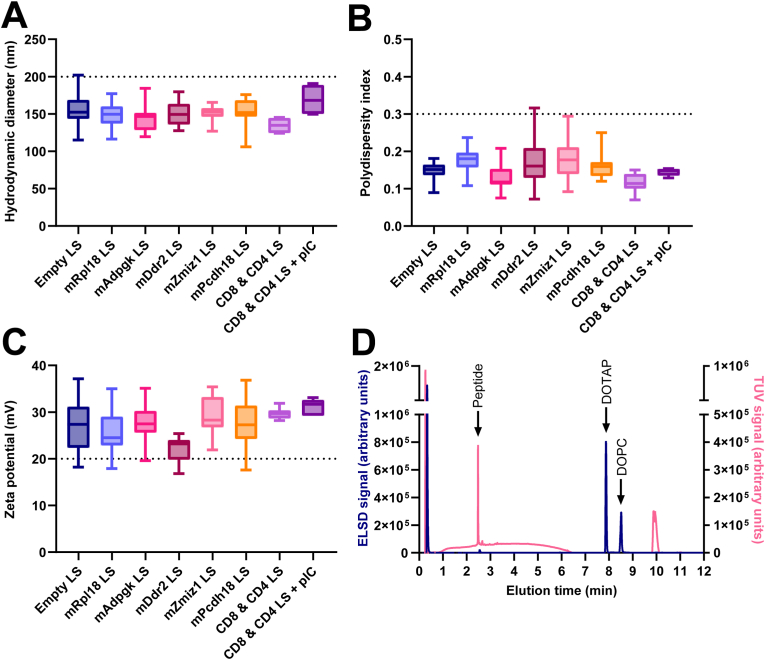
**Physicochemical characteristics of neoepitope-loaded cationic liposomes. (A)** Average hydrodynamic diameter, **(B)** average polydispersity index (PDI), and **(C)** average zeta potential of neoepitope peptide-loaded and empty cationic liposomes, as well as mixed CD8 & CD4 neoepitope-loaded liposomes, with or without added Poly(I:C) (pIC). Average hydrodynamic diameter and PDI were determined by dynamic light scattering (DLS), average zeta potential was determined by laser Doppler electrophoresis. Data shown as mean ± SD, n = 5-8 independent batches per liposomal formulation, measured in triplicates. **(D)** Representative UPLC chromatogram of a liposome-encapsulated peptide, showing the separation of the peptide (*mRpl18*) and lipids (DOTAP, DOPC) by ELSD (evaporative light scattering detector) and TUV (two-channel UV/vis detector) signals.

In our initial studies we screened seven MC38 neoepitopes (*mRpl18, mAdpgk, mDpagt, mReps1, mDdr2, mZmiz1,* and *mPcdh18*) for their potential to be formulated into cationic liposomes with our platform technology (Table 1, Table 2, Supplementary Table 1). Next, we selected five MC38 tumor-derived peptides to be formulated into liposomes as a personalized neoepitope vaccine [21,23] (Table 1), which were quantified by UPLC (Supplementary Fig. 1A) and extensively characterized. The synthetic peptide (SP) neoepitopes included naturally flanking sequences and were individually encapsulated in cationic liposomes. The resulting liposomal dispersions had comparable hydrodynamic diameters (Z-average: 146 - 155 nm) and polydispersity (PDI: 0.14 - 0.18), and were all positively charged (Zeta potential: 21.8 - 29.2 mV; Table 2, Fig. 1A–C). Cryo-EM imaging revealed that the particles in our formulations were predominantly bi-lamellar (Supplementary Fig. 1B). The different liposomal formulations remained stable upon storage for at least 8 weeks at 4 °C (Supplementary Fig. 1C–F).

**Table 1:** **MHC class I and MHC class II-presented MC38 neoepitopes.** Amino acid sequences, molecular weight, isoelectric point and Gravy index of selected MHC I/CD8 T cell and MHC II/CD4 T cell neoepitope peptides derived from MC38 tumor cells. Mutated amino acid residues are shown in red.

Peptide	Specificity	Length	Sequence	Molecular Weight	Isoelectric Point	Gravy Index
mRpl18	CD8/K^b^	18	KAGGKILTFD**R**LALESPK	1944.30 g/mol	10.34	−0.267
mAdpgk	CD8/D^b^	15	ELASMTN**M**ELMSSIV	1655.97 g/mol	3.09	+0.680
mDdr2	CD4/I-A^b^	23	SEASEWEP**H**AVYFPLVLDDVNPS	2601.81 g/mol	3.39	−0.304
mZmiz1	CD4/I-A^b^	17	RPPADFTQPAA**S**AAAAA	1612.76 g/mol	6.85	−0.035
mPcdh18	CD4/I-A^b^	16	SP**W**AYITTVTATDPDL	1750.92 g/mol	2.91	+0.006

**Table 2:** **Physicochemical characteristics of neoepitope-loaded cationic liposomes.** For all liposomal formulations, we determined the hydrodynamic diameter and polydispersity index of particles by dynamic light scattering (DLS) analysis, as well as their zeta potential by laser Doppler electrophoresis. Further, we evaluated the encapsulation efficiency, peptide and lipid (DOTAP and DOPC) recovery in the different liposomal dispersions by UPLC-TUV and UPLC-ELSD. We were unable to reliably quantify the encapsulation efficiency for *mZmiz1* due to interactions between the peptide and the Vivaspin columns. Data shown as mean ± SD, n = 5-8 independent measurements per formulation, N/D = not determined, N/A = not applicable.

Peptide	Hydrodynamic diameter (nm)	Polydispersity index	Zeta potential (mV)	Encapsulation efficiency (%)	Peptide recovery (%)	DOTAP recovery (%)	DOPC recovery (%)
mRpl18	149 ± 18	0.18 ± 0.03	25.8 ± 5.0	52.5 ± 11.6	86.4 ± 4.5	55.9 ± 16.8	45.6 ± 13.6
mAdpgk	146 ± 22	0.14 ± 0.04	27.6 ± 4.7	44.2 ± 21.8	73.7 ± 5.4	56.8 ± 23.9	45.6 ± 12.3
mDdr2	151 ± 17	0.17 ± 0.07	21.8 ± 3.1	98.6 ± 3.2	83.9 ± 0.4	64.5 ± 7.7	66.6 ± 12.4
mZmiz1	151 ± 11	0.18 ± 0.06	29.2 ± 4.1	N/D	78.3 ± 15.8	65.4 ± 8.1	67.1 ± 12.6
mPcdh18	150 ± 23	0.16 ± 0.04	27.7 ± 5.8	76.3 ± 19.0	76.4 ± 14.7	63.1 ± 14.6	54.7 ± 16.8
(Empty)	155 ± 23	0.15 ± 0.02	27.2 ± 5.6	N/A	N/A	52.7 ± 22.0	55.5 ± 15.7

For vaccination studies, these cationic liposomal formulations were adjuvanted with Poly(I:C). Due to the negative charge of Poly(I:C), we additionally evaluated the effects of electrostatic interactions between the liposomes and the adjuvant on the physicochemical characteristics of the vaccine formulation (Fig. 1A–C). Mixing the peptide loaded liposomes together did not lead to any significant changes in particle size or charge, compared to the individual peptide-loaded liposome formulations. Adding Poly(I:C) to this mixture slightly increased the particle size by 35 nm on average, but had no effects on the charge.

Peptides and lipids were separated by UPLC and detected and quantified by UV and evaporative light scattering (ELS). Peptides eluted around 2 to 4 min, depending on the specific peptide, DOTAP eluted at 8.00 min, DOPC at 8.25 min (Fig. 1D). The average encapsulation efficiency (44.2 - 98.6%) and peptide recovery (73.7 - 86.4%), as well as recovery of DOTAP (52.7 - 65.4%) and DOPC (45.6 - 67.1%), indicating effective loading of peptides in liposomes, and minimal peptide loss during the formulation process (Table 2). Peptide recovery and encapsulation efficiency appeared to depend on the physicochemical properties of the peptides, which is in line with previous studies investigating the encapsulation of peptides in DOTAP:DOPC liposomes [12].

### 2.2 *In vitro* uptake of liposome-encapsulated (neo)epitopes by DCs and activation of antigen-specific CD8 T cells

**Fig. 2:**
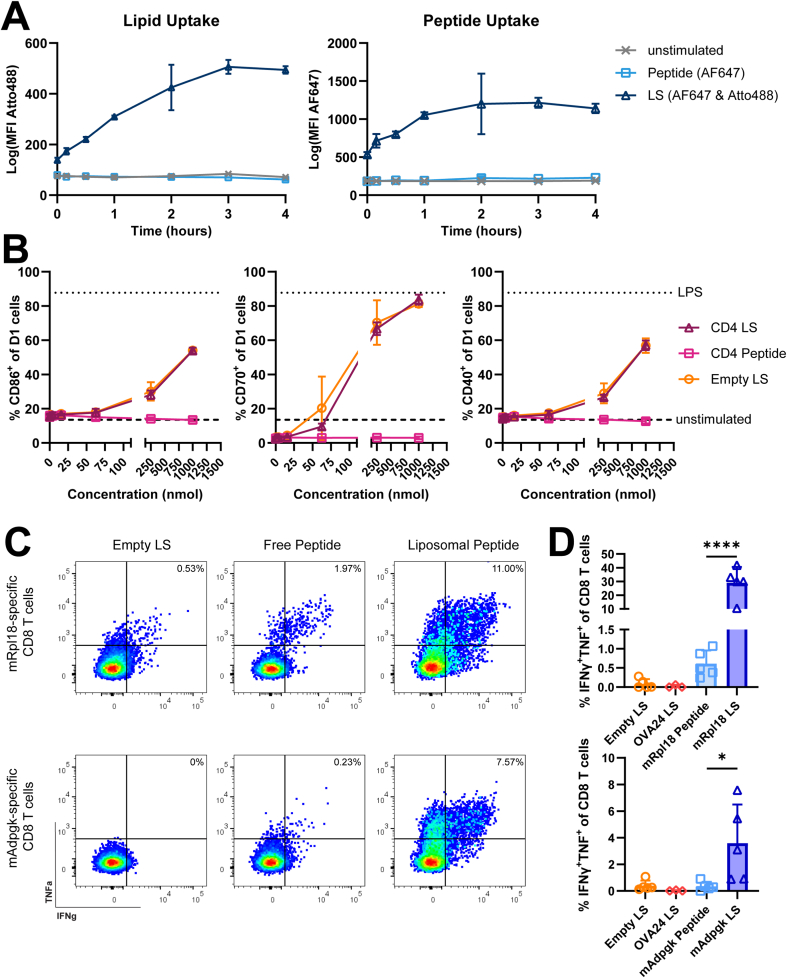
***In vitro* activation of dendritic cells and neoepitope-specific CD8 T cell lines. (A)** Murine D1 dendritic cells were incubated with free OVA24 peptide labelled with AF647, liposome-encapsulated OVA24 (peptide labelled with AF647, lipids labelled with Atto488), or were left unstimulated. Antigen uptake by DCs was determined by flow cytometry over the course of 4 h. Graphs show the uptake of Atto488-lipids (left) and of AF647-labelled peptide (right), quantified through the mean fluorescence intensity (MFI) of the fluorochromes. Data shown as mean ± SD, n = 3 samples per group and timepoint. **(B)** D1 cells were incubated with different concentrations of free CD4 neoepitope peptides (n = 6 samples), liposome-encapsulated neoepitopes (n = 6 samples), or empty liposomes (n = 2 samples). Graphs show the expression frequency of DC maturation markers CD86, CD70 and CD40 after 24 h. The dashes line represents a negative control (unstimulated cells), the dotted line represents a positive control (LPS-stimulated cells). Data shown as mean ± SD. **(C)** D1 cells were loaded with either free or liposome-encapsulated *mRpl18* and *mAdpgk* peptide, liposome-encapsulated OVA24 peptide, or empty liposomes (2.5 μM per peptide). DCs were co-cultured with *mRpl18-*or *mAdpgk*-specific CD8 T cell lines, followed by intracellular cytokine staining and flow cytometric analysis. Representative dot plots show IFNɣ^+^TNF^+^*mRpl18* (upper) and *mAdpgk*-specific CD8 T cells (lower) and **(D)** average frequencies of IFNɣ^+^TNF^+^ CD8 T cells. Data shown as mean ± SD, n = 5 samples per group, statistical significance was determined by one-way ANOVA with Tukey's multiple comparisons test.

The uptake of liposome-encapsulated peptide epitopes by dendritic cells (DCs) was evaluated using murine D1 cells. OVA24 peptide was fluorescently labelled with AF647 and added to the DCs either as free peptide or encapsulated in cationic liposomes, which contained an Atto488-labelled lipid. Over the course of 4 h, both the lipid and peptide were efficiently taken up by DCs incubated with liposome-encapsulated OVA24, whereas the free peptide was not taken up (Fig. 2A).

Furthermore, we investigated the effects of cationic liposomal formulation of neoepitope peptides on the maturation of DCs. Incubation of D1 cells with different concentrations of free neoepitope peptides, liposome-encapsulated neoepitope peptides or empty liposomes showed that both neoepitope-loaded and empty liposomes could induce the expression of maturation markers CD86, CD70 and CD40, while unencapsulated neoepitopes did not lead to maturation (Fig. 2B).

To determine the subsequent effects of liposomal neoepitope formulation on CD8 T cell activation *in vitro*, D1 cells were loaded with free or liposome-encapsulated *mRpl18* or *mAdpgk,* empty liposomes or liposome-encapsulated OVA24. The DCs were then co-cultured with two neoepitope-specific CD8 T cell lines, recognizing neoepitopes *mRpl18* and *mAdpgk* respectively. The CD8 T cells produced TNF and IFNγ in response to stimulation with the corresponding liposomal neoepitope, indicating the cationic liposomes were effectively taken up, the peptide antigens were processed and presented by the DCs leading to activation of the tumor-specific T cells (Fig. 2C and D). DCs incubated with empty liposomes, or liposomes loaded with irrelevant peptide OVA24 did not induce any response from the neoepitope-specific CD8 T cell lines (Fig. 2C and D).

### 2.3 *In vivo* priming of CD4 and CD8 T cells and immunogenicity of liposome-formulated neoepitopes

To determine the immunogenicity of the neoepitope-loaded cationic liposomes *in vivo*, naive mice were vaccinated on day 0 and day 14 with either soluble peptide or liposome-encapsulated peptide, adjuvanted with TLR ligand Poly(I:C).

**Fig. 3:**
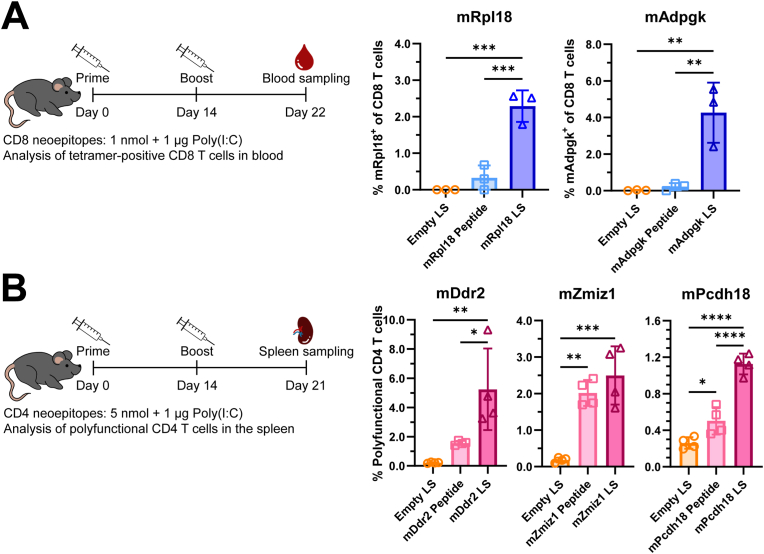
***In vivo* priming of CD8 and CD4 T cells by liposomal neoepitopes. (A)** To determine the immunogenicity of liposome-formulated CD8 neoepitopes, naive C57BL/6 mice were vaccinated in a prime-boost setting on days 0 and 14, followed by analysis of *mRpl18*-and *mAdpgk*-specific CD8 T cells on day 21. The vaccines contained either empty liposomes, a free peptide neoepitope or cationic liposomes containing a single neoepitope, at a dose of 1 nmol per peptide and 1 μg adjuvant Poly(I:C). Levels of neoepitope-specific CD8 T cells in the blood of vaccinated mice were determined on day 22 by staining with neoepitope-specific MHC I tetramers and flow cytometry (n = 3 mice per group). **(B)** To determine CD4 T cell priming by liposomal CD4 neoepitopes, naive C57BL/6 were vaccinated on days 0 and 14 with either empty liposomes, a mix of all free peptide neoepitopes or a mix of liposomes containing all neoepitopes, at a dose of 5 nmol per peptide and 1 μg adjuvant Poly(I:C). Mice were sacrificed on day 21, splenocytes were incubated with peptide-loaded DCs, followed by intracellular cytokine staining and flow cytometry to determine the activation of neoepitope-specific CD4 T cells. Graph shows polyfunctional CD4 T cells co-expressing at least two markers out of CD40L, IFNγ, IL-2 and TNF (n = 4 mice per group). Data shown as mean ± SD, statistical significance was determined by one-way ANOVA with Tukey's multiple comparisons test for A and B.

To study the responses to MHC class I neoepitopes *mRpl18* and *mAdpgk*, mice were vaccinated intradermally with 1 nmol (1.6 - 2.6 μg) of peptide, ±35 nmol (±25 μg) liposomes, and 1 μg Poly(I:C) per dose. In the case of both neoepitopes, induction of neoepitope-specific CD8 T cells in blood on day 22 was 3-5 fold higher in mice vaccinated with liposome-encapsulated peptide compared to mice vaccinated with soluble peptide (Fig. 3A). Furthermore, we found that administering a vaccine composed of a mixture of four liposome-encapsulated MHC class I neoepitopes (*mRpl18, mAdpgk, mDpagt, mReps1*) did not alter the efficiency of CD8 T cell priming compared to vaccination with individual liposomal formulations (Supplementary Fig. 2).

In the case of MHC class II neoepitopes *mDdr2, mZmiz1* and *mPcdh18*, naive mice were vaccinated on day 0 and 14 with 5 nmol soluble or liposome-encapsulated peptide, adjuvanted with 1 μg Poly(I:C). On day 21 the mice were sacrificed to assess the number of neoepitope-reactive CD4 T cells in the spleen. This was done by restimulating the splenocytes with *mDdr2, mZmiz1* or *mPcdh18* peptides. We found higher frequencies of polyfunctional CD4 T cells, positive for at least two markers out of CD40L, IL-2, IFNγ and TNF, in the splenocytes of mice vaccinated with cationic liposomes, compared to mice vaccinated with soluble peptides (Fig. 3B).

### 2.4 Enhanced neoepitope-specific CD8 T cell responses after vaccination with a cocktail of liposomal MHC class I and class II neoepitopes

**Fig. 4:**
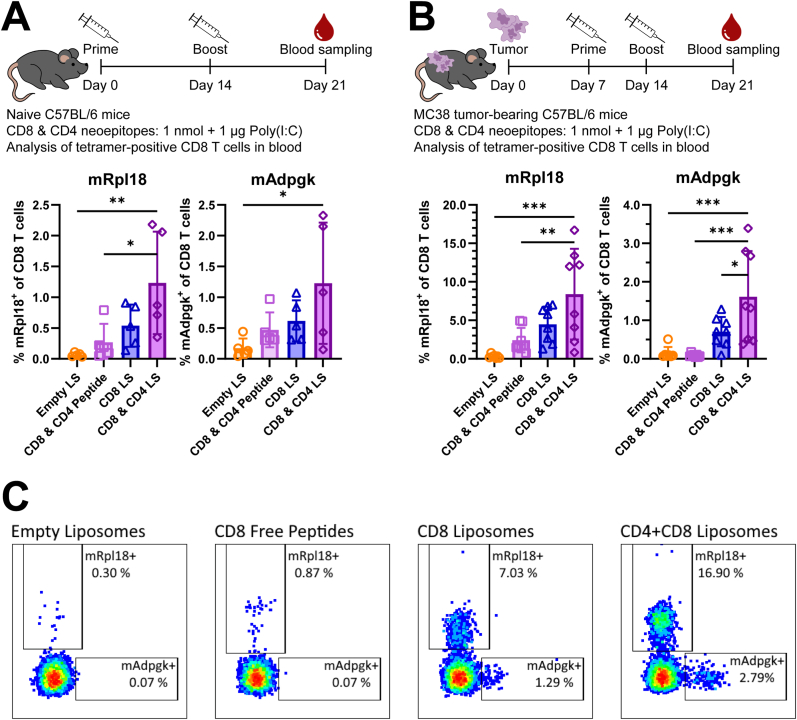
**Enhanced priming of neoepitope-specific CD8 T cells by liposomal vaccine containing MHC I and MHC II neoepitopes *in vivo*. (A)** Naive (n = 5 mice per group) and **(B)** MC38 tumor-bearing C57BL/6 mice (n = 8 mice per group) were vaccinated twice with either empty liposomes, free CD8 and CD4 peptide neoepitopes mixed with empty liposomes, liposome-encapsulated CD8 neoepitopes, or liposome-encapsulated CD8 and CD4 neoepitopes, all at a dose of 1 nmol per neoepitope and adjuvanted with a total of 1 μg Poly(I:C) (except for the “empty liposome” group). In both settings vaccination with liposome-encapsulated CD8 and CD4 neoepitopes led to the highest frequencies of *mRpl18* and *mAdpgk*-specific CD8 T cells in blood, as revealed by MHC I tetramer staining. Data shown as mean ± SD, statistical significance was determined by one-way ANOVA with Tukey's multiple comparisons test for A and B. **(C)** Representative dot plots showing neoepitope-specific CD8 T cell responses in the blood of MC38 tumor-bearing mice.

Next, we studied the priming of neoepitope-specific T cells by a liposomal vaccine containing all selected CD8 and CD4 neoepitopes. These neoepitopes were administered *in vivo* as an equimolar mixture of cationic liposome-encapsulated peptides. Vaccination of both naive (Fig. 4A) and MC38 tumor-bearing mice (Fig. 4B and C) with liposomal CD8 and CD4 neoepitopes resulted in at least twofold higher frequencies of *mRpl18* and *mAdpgk*-specific CD8 T cells in blood compared to vaccination with only liposomal CD8 neoepitopes. Thus, the addition of cationic liposome-encapsulated CD4 neoepitopes to CD8 neoepitopes significantly improved the priming of MC38 tumor-specific CD8 T cells. Furthermore, the encapsulated CD8 and CD4 neoepitopes outperformed the mixture of soluble peptide neoepitopes with empty liposomes (Fig. 4A–C). Considering these results, we evaluated the efficacy of our liposomal neoepitope vaccine in both prophylactic and therapeutic *in vivo* tumor studies.

### 2.5 Cationic liposomal neoepitope vaccination induced tumor control in prophylactic and therapeutic settings

**Fig. 5:**
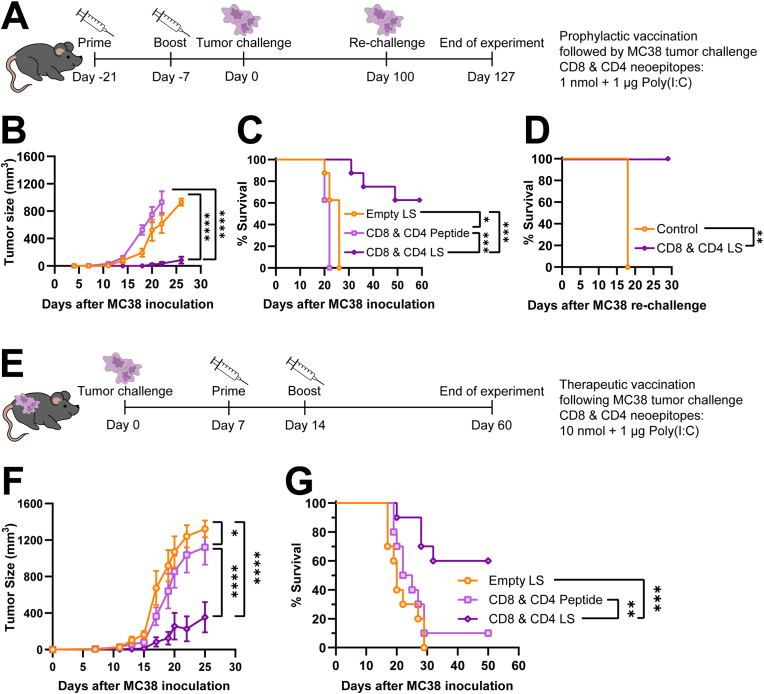
**Tumor control by cationic liposomal multi-neoepitope vaccination. (A)** Prophylactic treatment scheme; mice are vaccinated on days −21 and −7 with empty liposomes, free CD8 & CD4 neoepitopes mixed with empty liposomes (10 nmol per neoepitope, 1 μg Poly(I:C)), or liposome-encapsulated CD8 & CD4 neoepitopes (10 nmol per neoepitope, 1 μg Poly(I:C)). On day 0, mice are challenged with a lethal dose of MC38 cells, and outgrowth of tumors is observed. On day 100 the surviving mice were again re-challenged with MC38 cells. **(B)** Average tumor sizes per group after prophylactic vaccination (n = 8 mice per group, data shown as mean ± SEM, statistical significance determine by two-way ANOVA with Tukey's multiple comparisons test). **(C)** Survival curves showing the survival of mice challenged with MC38 after prophylactic vaccination with empty liposomes, free CD8 and CD4 neoepitope peptides mixed with empty liposomes, or liposomal CD8 and CD4 neoepitopes (n = 8 mice per group, statistical significance determined by Log-rank test). **(D)** Survival curves after re-challenge of surviving mice that were previously vaccinated with CD8 and CD4 neoepitope liposomes (n = 8 mice) or control mice (n = 6 mice) with MC38 tumor cells on day 100 after the original tumor challenge (statistical significance determined by Log-rank test). **(E)** Therapeutic treatment scheme; mice were challenged with a lethal dose of MC38 cells. When the tumors started to grow out, the animals were vaccinated twice (day 7 and day 14) with empty liposomes, free CD8 & CD4 neoepitopes with empty liposomes (10 nmol per neoepitope, 1 μg Poly(I:C)), or liposome-encapsulated CD8 & CD4 neoepitopes (10 nmol per neoepitope, 1 μg Poly(I:C)). **(F)** Average tumor sizes per group after therapeutic vaccination (n = 10 mice per group, data shown as mean ± SEM, statistical significance determine by two-way ANOVA with Tukey's multiple comparisons test). **(G)** Survival curves showing the survival of MC38 tumor-bearing mice that received therapeutic vaccination with empty liposomes, free CD8 and CD4 neoepitope peptides mixed with empty liposomes, or liposomal CD8 and CD4 neoepitopes (n = 10 mice per group, statistical significance determined by Log-rank test).

The functionality of the neoepitope-specific T cells induced by liposomal vaccination was evaluated by challenging vaccinated mice with MC38 tumor cells. The vaccines comprised 1 μg Poly(I:C) and peptide: 10 nmol each of two CD8 neoepitopes (*mRpl18, mAdpgk*) and three CD4 neoepitopes (*mDdr2, mZmiz1, mPcdh18*), either individually encapsulated in cationic liposomes, mixed with empty liposomes, or as free peptides. After vaccination, the mice were challenged with a lethal dose of MC38 tumor cells (Fig. 5A). MC38 tumors grew out rapidly in the groups receiving either only empty liposomes or peptide neoepitopes mixed with empty liposomes, resulting in all mice from these groups meeting the humane endpoint criteria within 25 days after tumor inoculation (Fig. 5B–C). Only three out of eight mice vaccinated with cationic liposome-encapsulated CD8 and CD4 neoepitopes developed MC38 tumors with delayed outgrowth, with the remaining five mice fully controlling tumor outgrowth and surviving the challenge (Fig. 5D). The surviving mice were re-challenged with MC38 100 days after the first tumor challenge and all survived until at least 30 days later (Fig. 5E), indicating that the mice developed a functional memory immune response against MC38 tumor cells.

Moreover, the liposomal vaccine was also tested in a therapeutic setting (Fig. 5F). Here mice were first inoculated with a lethal dose of MC38 tumor cells. After the tumors were established, mice were treated with the neoepitope liposomal vaccine on days 7 and 14. This resulted in a significant delay of MC38 tumor outgrowth in all mice (Fig. 5G), and led to complete regression of the tumors in 60% of the mice (Fig. 5H).

## 3 Discussion

In this study we report the development of a cationic liposomal neoepitope peptide-based cancer vaccine and its application in a colorectal cancer model for personalized cancer vaccination. MC38 tumor-derived MHC class I and class II neoepitope peptides with varying physicochemical properties could be encapsulated in DOTAP:DOPC liposomes. Liposome-encapsulated MHC class I epitopes improved the priming of tumor-specific CD8 T cells *in vitro* and *in vivo* compared to free peptides. Previously, the MC38-derived MHC class I neoepitopes *mRpl18* and *mAdpgk* have been shown to play a dominant role in anti-tumor CD8 T cell responses [21,25,26]. The more recently identified MHC class II neoepitopes *mDdr2, mZmiz1* and *mPcdh18* were shown to induce polyfunctional CD4 T cells and further improve vaccine efficacy when combined with the CD8 T cell epitopes [23,25]. These results are in line with current literature suggesting that both tumor-specific CD8 and CD4 T cells are required for optimal anti-tumor immunity [[27], [28], [29]]. Tumor-specific CD4 immunity appears not only important for improved CD8 T cell priming but can also act locally in the tumor microenvironment and associated lymphoid organs where antigen is present [25,30]. Here we show that neoepitope-specific T cells induced by vaccination were able to protect the mice against challenge with a lethal dose of MC38 tumor cells. Additionally, therapeutic vaccination of MC38 tumor-bearing mice with liposomal MHC class I and II neoepitopes was able to control the outgrowth of tumors and cure 60% of tumor-bearing mice. The capacity of the liposomal vaccine to accommodate and efficiently deliver both MHC class I and MHC class II neoepitopes *in vivo* shows great potency for developing personalized cancer vaccines.

For all neoepitopes evaluated in this study, both MHC class I and class II, neoepitope peptides encapsulated in cationic liposomes outperformed unencapsulated peptides regarding the priming of functional CD8 and CD4 T cells. Prior to that, we demonstrated that liposomal formulation improved the uptake of peptides by APCs and led to DC maturation. Furthermore, our data implies that there are electrostatic interactions between the cationic liposomes and the anionic Poly(I:C), leading the adjuvant to adsorb to the liposome surface.

Our results are in line with previous studies, which suggest that liposomal formulation likely improves the uptake of peptide antigen and adjuvants (in this case DOTAP and Poly(I:C)) by the same dendritic cells, leading to optimal T cell priming, even when the adjuvant (Poly(I:C:)) is not co-encapsulated alongside the antigen [11,31,32]. We observed that vaccination with empty liposomes mixed with free peptides was more potent than free peptides only, but vaccination with liposome-encapsulated peptides was superior in terms of generating neoepitope-specific T cells [11,33].

Cationic adjuvant formulation is a platform in which antigenic proteins are admixed with cationic particles [34,35], thereby showing good immunogenicity. However, we show that in the case of peptide antigens, encapsulation is crucial for eliciting effective T cell immune responses and tumor control. Efficient uptake of the synthetic peptide antigen by dendritic cells is crucial for effective T cell priming, and therefore optimal antigen encapsulation is key. Vaccine efficacy is influenced by liposome composition, antigen format (e.g., peptide, protein, cell lysate, etc.), adjuvants, dosage and administration; thus, further improvements could be made to the formulation we currently employ.

Other platforms and nanoscale delivery systems have recently been evaluated in the MC38 tumor model, which aim to co-deliver neoepitopes and adjuvants to the same APC in order to induce potent anti-tumor immune responses. For example, synthetic peptide epitopes conjugated to TLR7/8a ligands self-assembling into nanoparticles (20 nm) have been evaluated as a prophylactic vaccine against MC38 tumors. Vaccinated mice displayed delayed outgrowth of MC38 tumors, but the vaccine did not increase overall survival [36]. In another study, synthetic high-density lipoprotein nanodiscs (10 nm) loaded with antigenic peptides and TLR9 ligand CpG led delayed the outgrowth of established MC38 tumors, or to complete tumor regression when combined with anti-PD-1 immunotherapy [37,38], which MC38 tumors have been shown to be sensitive to Refs. [39,40]. Montanide is a regularly used adjuvant, but is only minimally effective by comparison. In a previous study, we found that the peptide dose can be reduced a 65-fold utilizing cationic liposomes instead of Montanide with CpG, while leading to superior T cell responses and tumor control [11]. In our present study, we found that therapeutic vaccination with cationic liposome-encapsulated neoepitopes significantly delayed the outgrowth of MC38 tumors and led to complete tumor regression in 60% of all mice even without additional immunotherapeutic interventions such as checkpoint blockade. Combining liposomal multi-neoepitope vaccination with immune checkpoint therapy could further improve survival and essential to combine in treatment of tumors with immunosuppressive microenvironments [41].

Cationic lipid delivery platforms are currently widely used for mRNA-based vaccines, and recent studies show great promise for therapeutic mRNA neoepitope cancer vaccines. Neoantigenic mRNA formulated in lipid nanoparticles and lipopolyplexes has been successfully used to alleviate tumor burden in several murine preclinical cancer models, including MC38 [42,43], and was shown to contribute to improved T cell responses and recurrence-free survival in human pancreatic cancer patients [44,45]. While both peptide and mRNA vaccines come with their unique advantages and drawbacks, one of the most relevant considerations for therapeutic cancer vaccines is dosing. It has been shown that while the efficacy of mRNA vaccines also depends on the administered dosage [43], the dosing is much harder to control compared to peptide-based vaccines. This is especially relevant in light of recent studies showing that the ratio of MHC I to MHC II neoepitopes in cancer vaccines influences their overall efficacy [46,47]. Different ratios between the individual neoepitopes within a vaccine are more easily achieved using individually encapsulated peptides rather than mRNA encoding for multiple neoepitopes at once.

In contrast, several studies suggest that co-delivery of MHC class I and class II epitopes to the same APC results in superior priming of CD8 and CD4 T cells and support T cell help provided by CD4 to CD8 T cells [28,48,49]. While we are able to encapsulate a wide range of physicochemically diverse peptides in DOTAP:DOPC liposomes by thin film dehydration-rehydration, the encapsulation efficiency varies from peptide to peptide due to these differences in charge and hydropathy. This would influence dosing accuracy. A relevant advantage of this liposomal platform is its simple particle composition and the feasibility of the production process. The platform consists only of two lipids, DOTAP and DOPC, and a synthetic peptide, which is then mixed with a clinically used adjuvant Poly(I:C). Additionally, we show that the liposomal formulations produced using this methods remain stable over the course of several weeks. Previously we have reported the optimization of peptide loading which allows loading of a wide range of physicochemically diverse peptides [12], resulting in a rapid production process yielding liposomes with consistent particle sizes below 200 nm and allowing for sterile filtration of the vaccine after production. In this study we were able to show that this process can be applied to produce effective therapeutic vaccines using tumor-derived neoepitopes, requiring only minor fine-tuning. For example, we found that *mPchd18* is not soluble in aqueous solutions and instead opted to use an organic solution to incorporate the peptide into the liposomes.

To conclude, we describe a cationic liposomal platform for the efficient encapsulation of neoepitope peptides and demonstrate that these formulations significantly improve the efficacy of peptide-based personalized cancer vaccines. Our results implicate that cationic peptide-based neoantigen formulations can be used to improve personalized, tumor-specific immunotherapy. In future studies, we hope to further delineate the effects liposomal formulation of cancer vaccines on T cells and the tumor microenvironment.

## 4 Material & methods

### 4.1 Materials

Synthetic peptides (SPs) comprising the MC38-L-derived [24] MHC I neoepitopes (*mRpl18, mAdpgk*) and MHC II neoepitopes (*mDdr2, mZmiz1, mPcdh18*), as well as OVA24, containing the chicken ovalbumin-derived CD8 T cell epitope SIINFEKL, and the fluorescent AF647-OVA24 conjugate were synthetized and purified at the peptide facility of the Department of Immunology at the Leiden University Medical Center (“LUMC”; Leiden, The Netherlands). In all MC38-derived SPs of 15-23 amino acids, the minimal epitopes are surrounded by the natural flanking regions (Table 1) [21,23,26] Fluorescently labelled MHC I tetramers, mAdpgk-PE (Db-ASMTNMELM-PE) and mRpl18-APC (Kb-KILTFDRL-APC), were produced and purified at the peptide facility of the LUMC.

Lipids Dioleoyl-3-trimethylammonium propane (DOTAP) and 1,2-dioleoyl-sn-glycero-3-phosphocholine (DOPC) were purchased from Avanti Polar Lipids (Alabaster, Alabama, USA). Chloroform (CHCl_3_). The Atto488-labelled lipid 1,2-Dioleoyl-sn-glycero-3-phosphoethanolamine (Atto488-DOPE), trifluoroacetic acid (TFA) and ammonium hydroxide (NH_4_OH) purchased from Sigma-Aldrich (Burlington, Massachusetts, USA). Methanol (MeOH) and acetonitrile (AcN) were purchased from Biosolve (Valkenswaard, The Netherlands). Water (MQ) was treated with the Milli-Q® ultra-pure water purification system (Merck Millipore; Burlington, Massachusetts, USA).

Low molecular weight polyiosine-polycytidylic acid (Poly(I:C)) was purchased from InvivoGen (San Diego, California, USA).

### 4.2 Formulation of cationic liposomes

SP-loaded cationic liposomes were prepared using the thin film dehydration-rehydration method as described earlier [12,33]. In brief, both lipids were dissolved in chloroform and mixed at a 1:1 M ratio in a round bottom flask, followed by rotary evaporation in order to obtain a dry lipid film. Individual SPs were dissolved at 1 mg/ml and were added to the chloroform-dissolved lipids prior to rotary evaporation in CHCl_3_:MeOH:water (60:36:4, v/w; neoepitope *mPcdh18*) or were and added to the dry lipid film after rotary evaporation dissolved in 0.04% NH_4_OH (all other neoepitopes). The dry lipid film was hydrated with water or SPs dissolved at 1 mg/ml in water. The lipid-SP dispersion was then snap frozen and freeze dried. The resulting lipid-SP cake was slowly rehydrated with a phosphate buffer (10 mM, pH 7.4), followed by extrusion through 400 and 200 nm polycarbonate filters (Nucleopore Millipore, Kent, UK). Liposomes were purified and concentrated by centrifugation in vivaspin 2 columns (Sartorius Stedim Biotech GmbH, Göttingen, Germany; MW cut-off 300 kDa).

### 4.3 Physicochemical analysis

Hydrodynamic diameter and polydispersity index (PDI) of cationic liposomal formulations were determined by dynamic light scattering (DLS) using a Zetasizer Nano (Malvern Panalytical, Malvern, UK). Zeta potential of liposomes was determined by laser-Doppler-electrophoresis on a Zetasizer Nano. Liposome samples were diluted 1:75 in phosphate buffer for measuring.

### 4.4 Peptide and lipid recovery

The recovery of SP in the final liposome formulation was determined as previously described through reversed phase UPLC-UV analysis on a C18-1.7 μm (2.1 × 50 mm) column [50]. Lipid recovery was determined through reversed phase UPLC (Acquity UPLC system, Waters Corporation, Milford, Massachusetts, USA) coupled with an Evaporative Light Scattering Detector (ELSD). An ACN/MQ with 0.1% TFA gradient with a flow rate of 0.5 ml/min was used and the peptides were detected by measuring the UV absorbance at λ = 214 nm, lipids were measured on the ELSD. Quantification was done by integration of the area under the curve of the calibration lines by using MassLynx software (Waters Corporation, software 4.2). Peptide and lipid recovery were calculated through the following formula:

$$\text{Recovery}\;\left(\%\right)=\frac{Lipid/peptide\;in\;formulation}{Total\;added\;lipid/peptide}\;x\;100$$

### 4.5 Encapsulation efficiency

Total amount of peptide in the liposome formulation (prior to any further concentration or purification) and the free peptide measured in the flow through of Vivaspin 2 columns were determined through reversed phase UPLC-UV analysis and quantified using MassLynx software. Due to interactions between the Vivaspin columns and *mZmiz1*, we were unable to quantify the encapsulation efficiency of this formulation and instead dosed the formulation based on its total peptide content.

Efficiency of the encapsulation of peptides in cationic liposomes was determined using the formula:

$$\text{Efficiency}\;\left(\%\right)\text{ = }\frac{Total\;peptide\;-\;free\;peptide}{Total\;peptide}\;x\;100$$

### 4.6 DC uptake and maturation

D1 cells are a long-term growth factor-dependent immature splenic DC line derived from C57BL/6 mice. D1 cells were cultured as previously described [51]. To evaluate the effects of liposome-encapsulation on the uptake of peptide epitopes by DCs, D1 cells were incubated with AF647-labelled free peptide or AF647-labelled peptide encapsulated in Atto488-labelled cationic liposomes. Fluorescent liposomes contained 1% Atto488-labelled lipid DOPE. The uptake was measured by flowcytometry at different timepoints (0 to 4 h of incubation at 37 °C).

DC maturation was determined through the expression of maturation markers CD86 (clone B7.2, FITC), CD70 (clone FR70, PE-Cy7) and CD40 (clone 3723, APC) by D1 cells after incubation with different concentrations of peptide or liposomes (0.61-1000 nmol) for 24 h at 37 °C. Unstimulated D1 cells were used as a negative control, LPS-stimulated (2 μg/ml) D1 cells as a positive control.

### 4.7 *In vitro* antigen presentation assay

For antigen presentation assays, D1 cells were loaded overnight with the MC38-derived neoepitope peptides. T cell bulk lines specific for the neoepitopes *mRpl18* and *mAdpgk* [24] were added to the peptide-loaded D1 cells and co-cultured for 5 h in the presence of Brefeldin A (2 μg/ml), followed by intracellular cytokine staining. Samples were acquired using a BD LSR-II flow cytometer (BD Biosciences, Franklin Lakes, New Jersey USA) and frequencies of cytokine-producing neoepitope-specific CD8 T cells were determined using FlowJo 10.0 software (FlowJo LLC, Ashland, Oregon, USA).

### 4.8 Mice

Female C57BL/6 mice were obtained from Charles River (l’Arbresle, France) and Janvier (Le Genest-Saint-Isle, France) and were maintained under specific pathogen-free conditions. All animal experiments were performed in accordance with the guidelines and recommendations of the Dutch Animal Ethics Committee, the Dutch Experiments on Animals act and the LUMC. All individual experiments were approved by the Animal Welfare Body of the LUMC (permit AVD11600202013796).

### 4.9 Vaccination and tumor experiments

Mice were vaccinated with 1 to 10 nmol per liposome-encapsulated or free neoepitope peptide, according to the schedules described in the experiments. Per injection dose, the vaccines were admixed with 1 μg Poly(I:C). The dosing of liposome-formulated peptides was calculated based on the peptide-content measured after formulation, and was determined separately for each batch. For vaccination with free neoepitope peptides mixed with empty liposomes, the dose of empty liposomes was matched to the average lipid content applied in the liposomal vaccine groups (plus 1 μg Poly(I:C) per dose).

For prophylactic and therapeutic tumor experiments, 3 × 10^5^ MC38-L [24] tumor cells in PBS were injected subcutaneously in the right flanks of the mice. For therapeutic experiments, mice were confirmed to have a palpable tumor starting to form by day 7 after tumor cell injection, after which they were randomly assigned to experimental groups prior to being vaccinated. Animals were monitored closely and sacrificed upon tumor volume exceeding 1500 mm^3^ (volume = (length x width x height)/2). Further humane endpoint criteria for these experiments included significant weight loss (>20% of maximum recorded weight or >15% within the span of one week) or tumor necrosis.

### 4.10 *Ex vivo* analysis of T cell responses

Frequencies of neoepitope-specific CD8 and CD4 T cells were determined in peripheral blood and spleens of vaccinated mice. Blood samples were treated with erylysis buffer (NH_4_Cl 8.4 g/l, KHCO_3_ 1 g/l, pH = 7.4 ± 0.2; Apotheek AZL, Leiden, The Netherlands) and spleen samples were processed to single cell suspensions. Single cell suspensions were either stained directly or incubated with peptide-loaded D1 cells in the presence of 5 h, as described above. Data was acquired using BD LSR-II and Aurora 5L flow cytometers, and analyzed using FlowJo and OMIQ software (Dotmatics, Boston, Massachusetts, USA). The gating strategies applied for the analysis CD8 and CD4 T cell responses can be found in Supplementary Fig. 3A and 3B, respectively.

### 4.11 Statistical analysis

Statistical analysis was performed using GraphPad Prism 10.2.3 (GraphPad Software, Boston, MA,USA). Data are shown as mean +SD or mean +SEM depending on the experiment. Differences were considered statistically significant at p < 0.05. Multiplicity-adjusted P values are depicted in the figures as follows: ∗P ≤ 0.05, ∗∗P ≤ 0.01, ∗∗∗P ≤ 0.001, and ∗∗∗∗P ≤ 0.0001.

## CRediT authorship contribution statement

**Felicia S. Spitzer:** Conceptualization, Data curation, Formal analysis, Investigation, Methodology, Validation, Visualization, Writing – original draft, Writing – review & editing. **Jeroen Heuts:** Conceptualization, Formal analysis, Funding acquisition, Investigation, Methodology, Validation, Visualization, Writing – original draft. **Brett J. Hos:** Formal analysis, Investigation, Methodology, Validation, Visualization, Writing – review & editing. **Stefan Romeijn:** Conceptualization, Methodology, Resources, Validation. **Marcel G.M. Camps:** Data curation, Investigation, Validation, Writing – review & editing. **Ferry Ossendorp:** Conceptualization, Funding acquisition, Supervision, Writing – original draft, Writing – review & editing. **Koen van der Maaden:** Conceptualization, Funding acquisition, Methodology, Project administration, Supervision, Validation, Writing – original draft, Writing – review & editing.

## Declaration of competing interest

The authors declare the following financial interests/personal relationships which may be considered as potential competing interests: Jeroen Heuts reports financial support was provided by Leiden University. Koen van der Maaden, Ferry Ossendorp reports financial support was provided by Dutch Research Council. Koen van der Maaden, Ferry Ossendorp reports was provided by Oncode Accelerator. Koen van der Maaden reports a relationship with uPATCH B.V. that includes: equity or stocks. If there are other authors, they declare that they have no known competing financial interests or personal relationships that could have appeared to influence the work reported in this paper.

## Data Availability

Data will be made available on request.
