# Supplementary material for: Vaccination with cationic liposome-encapsulated CD4 and CD8 T cell neoepitopes induces superior tumor control
Source: Mater Today Bio. 2026 Apr 5;38:103103. doi: 10.1016/j.mtbio.2026.103103 (PMC13092067; doi:10.1016/j.mtbio.2026.103103)
Supplement: Multimedia component 1 [file mmc1.docx]

# Vaccination with cationic liposome-encapsulated CD4 and CD8 T cell neoepitopes induces superior tumor control

Felicia S. Spitzer^1^, Jeroen Heuts^1,2^, Brett J. Hos^1^, Stefan Romeijn^2^, Marcel G. M. Camps^1^, Ferry Ossendorp*^1^ & Koen van der Maaden*^1^^†^

^1^ Department of Immunology, Leiden University Medical Center, Leiden, The Netherlands

^2^ Division of BioTherapeutics, Leiden Academic Centre for Drug Research (LACDR), Leiden University, Leiden, The Netherlands

* Shared last authors

^†^ Corresponding author

## Supplementary Material

### Supplementary table 1

| **Peptide** | **Hydrodynamic diameter (nm)** | **Polydispersity index** | **Zeta potential (mV)** | **Encapsulation efficiency (%)** | **Peptide recovery (%)** | **DOTAP recovery (%)** | **DOPC recovery (%)** |
| --- | --- | --- | --- | --- | --- | --- | --- |
| mReps1 | 138 ± 3 | 0.20 ± 0.04 | 36.4 ± 5.5 | N/D | 3.7 ± 0.9 | N/D | N/D |
| mDpagt | 159 ± 5 | 0.21 ± 0.01 | 29.3 ± 4.0 | N/D | 12.3 ± 6.9 | N/D | N/D |

**Supplementary table 1: Physicochemical characteristics of cationic liposomes encapsulating MC38 neoepitopes.** Average hydrodynamic diameter, polydispersity index and zeta potential or cationic liposomes encapsulating MC38 neoepitope peptides mDpagt and mReps1. Data shown as mean ± SD, n=3 independent batches measured in triplicates, N/D = not determined.

### Supplementary figure 1


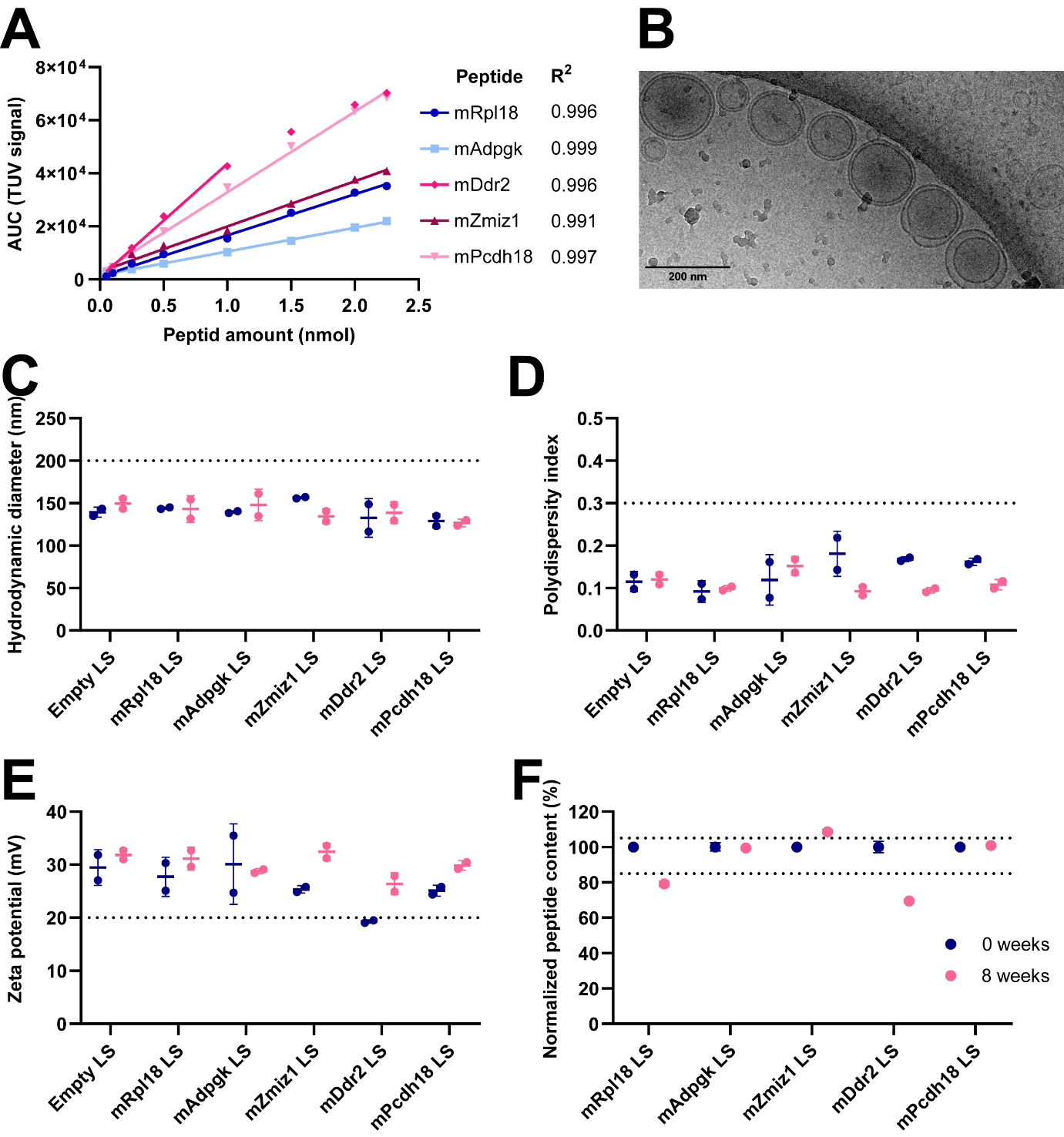


**Supplementary figure 1: Quantification, morphology and stability of neoepitope-loaded liposomes. (A)** Representative UPLC calibration curves for the quantification of neoepitope peptides *mRpl18, mAdpgk, mDdr2, mZmiz1,* and *mPcdh18*. All peptide calibration curves showed high linearity (R^2^ > 0.99) with a linear range of 0.05 – 2.25 nmol, with the exception of *mDdr2*, which was linear in a range of 0.05 – 1.00 nmol. Lower limit of quantification (LLOQ) was assumed as the lowest calibration point within linear range (0.05 nmol). Similarly, the upper limit of quantification (ULOQ) was defined as the highest point within the linear range. **(B)** Representative cryo-EM micrograph of cationic liposomes prepared by thin film dehydration-rehydration. The particles were predominantly bi-lamellar with two lipid bilayers. **(C)** Stability of independently prepared liposome batches immediately after production (0 weeks) and after 8 weeks of storage at 4°C (8 weeks). Hydrodynamic diameter and **(D)** polydispersity index were determined by DLS, **(E)** zeta potential was determined by laser Doppler electrophoresis. Data shown as mean ± SD, n=2 independent batches per formulation, measured in triplicates. **(F)** Stability of peptides in a liposome batch showing the peptide content in the formulation immediately after production and after 8 weeks of storage at 4°C (n=1 batch measured in triplicates).

### Supplementary figure 2


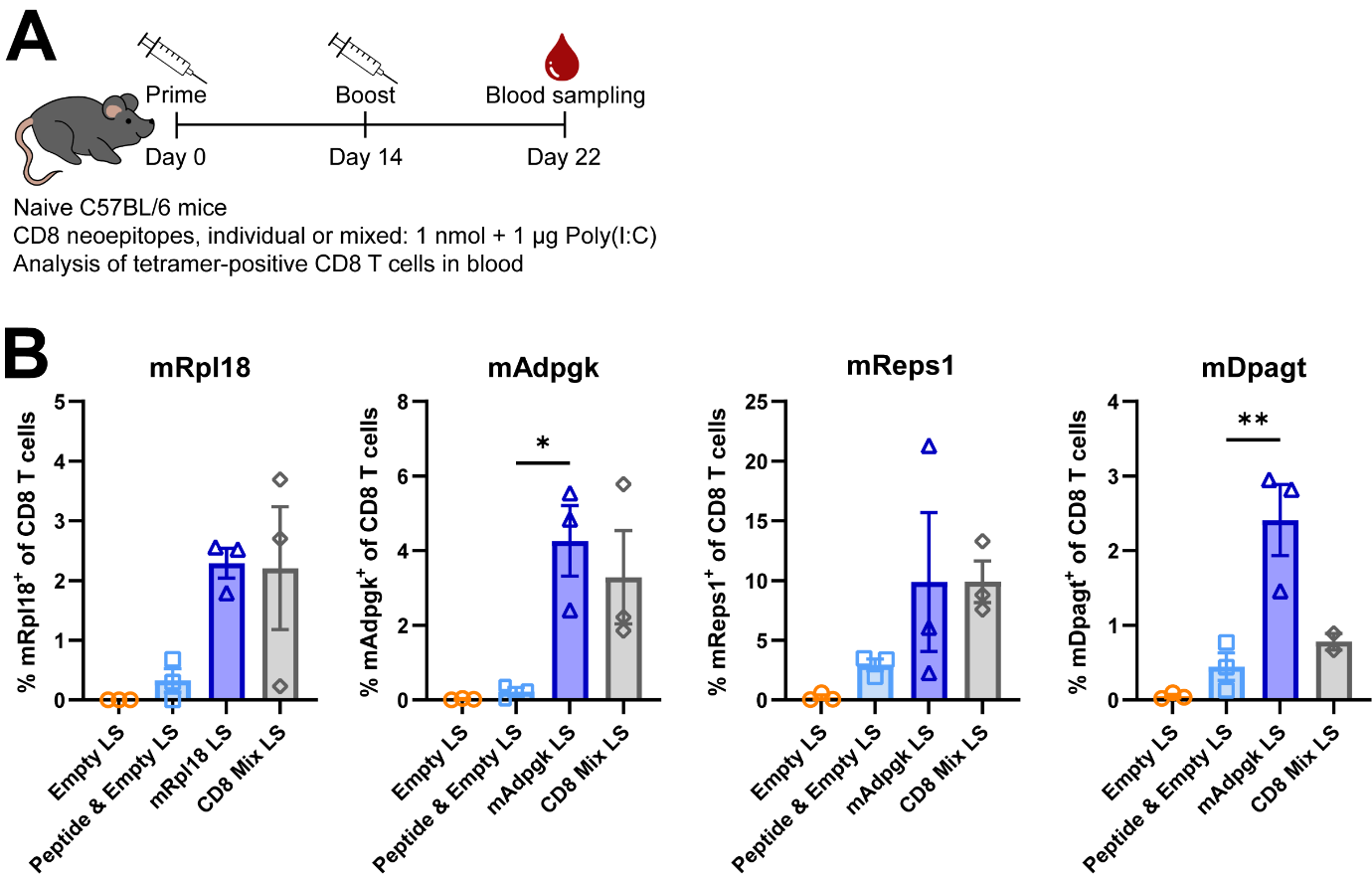


**Supplementary figure 2: *In vivo* priming of CD8 T cells after vaccination with liposomal neoepitope cocktail.** Naive C57BL/6 mice were vaccinated with either empty liposomes, an individual free neoepitope peptide (*mRpl18, mAdpgk, mReps1* or *mDpagt*) mixed with empty liposomes, a single MC38-derived neoepitope encapsulated in liposomes or with a mix of all four liposomal neoepitopes. **(A)** Mice were vaccinated on days 0 and 14 with a dose of 1 nmol per peptide, all vaccines were adjuvanted with 1 µg poly(I:C). **(B)** Levels of neoepitope-specific CD8 T cells in blood were determined on day 22 by flow cytometry. Data shown as mean ± SEM, n=3 mice per group, statistical significance was determined by one-way ANOVA with Tukey’s multiple comparisons test.

### Supplementary figure 3


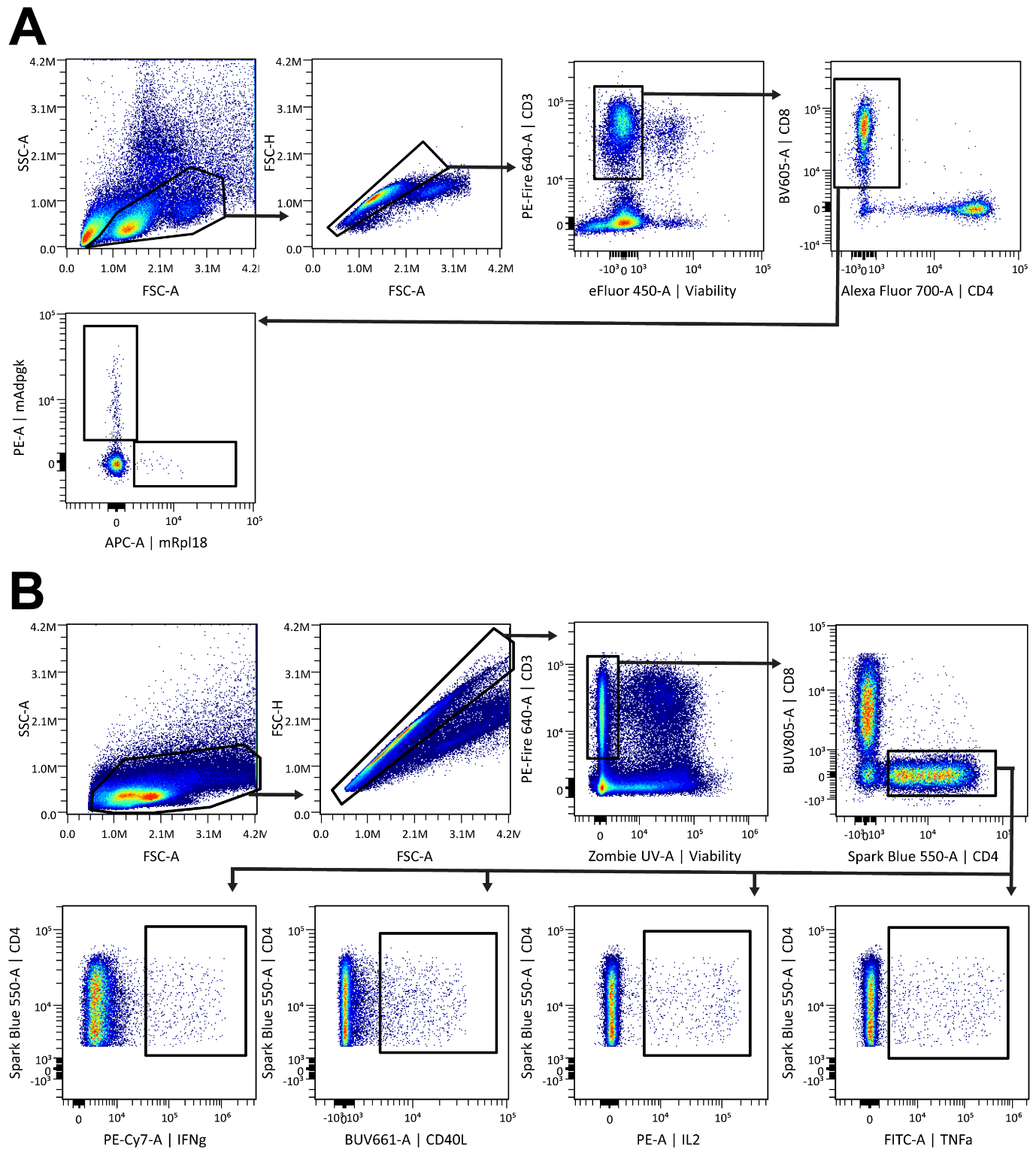


**Supplementary figure 3: Flow cytometry gating strategies. (A)** Example for the gating strategy applied to identify mRpl18 and mAdpgk-specific CD8 T cells using MHC I tetramers. **(B)** Example for the gating strategy used to identify cytokine-expressing CD4 T cells after peptide-restimulation of samples.
